# The universe and I: An exploration of the self and our place in the world

**DOI:** 10.36834/cmej.70714

**Published:** 2020-08-06

**Authors:** Nancy Duan

**Affiliations:** 1 University of British Columbia, British Columbia, Canada

**Figure:**
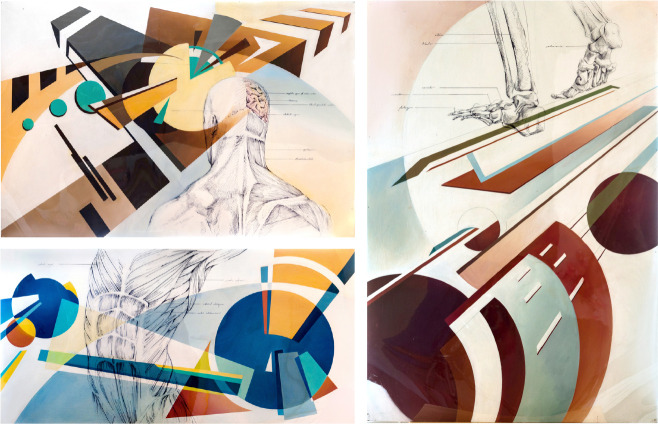


The universe and I is a triptych of paintings that aims to explore the relationship of the individual to the wider world around them. How do we reconcile the disconnect we feel between the self and the universe? In solipsistic fashion, these pieces represent the self as a concrete, physical, almost scientific entity, whereas the space around the self is evocative and abstract. It asks questions about self-discovery and the search for identity that are universal human experiences. The Universe and I: Part 1 focuses on the mind, conception, and creat-ivity. The Universe and I: Part 2 explores ideas about personal journeys and endurance in the face of opposition. The Universe and I: Part 3 depicts power, control, and finally achieving a strong sense of self.

These paintings were inspired by the visual juxtaposition of anatomical illustrations with modern abstraction. They were created with acrylic paint on wood panels and ink on acrylic glass overlay. Art is a powerful tool to convey the ineffable, and is always an exercise of empathy, whether between the artist and the viewer or between one viewer and another. Though the description provided is meant to help the viewer interpret these pieces, I invite you to create your own meanings through the intellectual, emotional, and physical reactions the artworks evoke in you.

